# Predicting the Dynamics of the COVID-19 Pandemic in the United States Using Graph Theory-Based Neural Networks

**DOI:** 10.3390/ijerph18073834

**Published:** 2021-04-06

**Authors:** Mohammad Reza Davahli, Krzysztof Fiok, Waldemar Karwowski, Awad M. Aljuaid, Redha Taiar

**Affiliations:** 1 Department of Industrial Engineering and Management Systems, University of Central Florida, Orlando, FL 32816, USA; fiok@ucf.edu (K.F.); wkar@ucf.edu (W.K.); 2 Department of Industrial Engineering, College of Engineering, Taif University, P.O. Box 11099, Taif 21944, Saudi Arabia; amjuaid@tu.edu.sa; 3 Department of Sports Sciences, MATIM, Université de Reims Champagne-Ardenne, 51100 Reims, France; redha.taiar@univ-reims.fr

**Keywords:** artificial intelligence, COVID-19 pandemic, graph neural networks, time series analysis

## Abstract

The COVID-19 pandemic has had unprecedented social and economic consequences in the United States. Therefore, accurately predicting the dynamics of the pandemic can be very beneficial. Two main elements required for developing reliable predictions include: (1) a predictive model and (2) an indicator of the current condition and status of the pandemic. As a pandemic indicator, we used the effective reproduction number (Rt), which is defined as the number of new infections transmitted by a single contagious individual in a population that may no longer be fully susceptible. To bring the pandemic under control, Rt must be less than one. To eliminate the pandemic, Rt should be close to zero. Therefore, this value may serve as a strong indicator of the current status of the pandemic. For a predictive model, we used graph neural networks (GNNs), a method that combines graphical analysis with the structure of neural networks. We developed two types of GNN models, including: (1) graph-theory-based neural networks (GTNN) and (2) neighborhood-based neural networks (NGNN). The nodes in both graphs indicated individual states in the United States. While the GTNN model’s edges document functional connectivity between states, those in the NGNN model link neighboring states to one another. We trained both models with R_t_ numbers collected over the previous four days and asked them to predict the following day for all states in the United States. The performance of these models was evaluated with the datasets that included R_t_ values reflecting conditions from 22 January through 26 November 2020 (before the start of COVID-19 vaccination in the United States). To determine the efficiency, we compared the results of two models with each other and with those generated by a baseline Long short-term memory (LSTM) model. The results indicated that the GTNN model outperformed both the NGNN and LSTM models for predicting Rt.

## 1. Introduction

On 8 December 2019, China announced treating several new virus cases due to a new strain of SARS (SARS-CoV-2), identified as coronavirus disease 2019 (COVID-19) [1]. Since then, COVID-19 has grown into a pandemic and spread to many countries. The US reported its first case of COVID-19 on 20 January 2020; since then, the pandemic has had unprecedented social and economic consequences [2]. In this dire situation, accurately predicting the dynamics of the pandemic can be very useful. To find the answer to this question, it will be important to select: (1) a reliable indicator of the condition and status of the pandemic at any given time and (2) an accurate prediction model.

One of the strongest indicators of the status of a pandemic is the effective reproduction number, or R_t_, which is defined as the number of new infections transmitted by a single contagious individual in a population that may no longer be fully susceptible [3]. This indicator, which both situation- and time-specific, is used to study changes in pathogen transmission over time. These changes may be the result of the implementation of different policies and/or changes in the overall immunity of the population, among other factors [4]. To bring the pandemic under control, the R_t_ needs to be less than 1 and as close as possible to 0. Therefore, an assessment of R_t_ over time may provide explicit feedback on the dynamics of the COVID-19 pandemic.

Calculating accurate values for R_t_ remains a challenging task. Two main methodologies have been used to calculate R_t_, including: (1) the case reproductive number and (2) the instantaneous reproductive number [3]. The first method calculates transmission within a specific cohort of people, while the second method calculates transmission at a specific point in time. Gostic et al. [3] reported that the instantaneous reproductive number is a more accurate method for estimating R_t_. Therefore, we used this method to calculate R_t_ in this study.

For an accurate prediction model, we select graph neural networks (GNNs) because: (1) this model considers the impact of states on each other, and (2) this method also has high efficiency when used for time series forecasting [5]. A GNN combines graphs with data structures that include nodes and edges as their two main components and a neural network architecture. We consider two types of GNN models, including the graph theory-based model (GTNN) and neighborhood-based model (NGNN). In both graphs, the nodes represent individual states within the US. However, the edges in the GTNN model represent the high correlation between time series of confirmed cases of COVID-19 (i.e., functional connectivity) while the edges in NGNN represent neighboring states. In both models, each node learns embedding information from the nodes to which it is connected.

Here, we developed an approach that can be used to predict the dynamics of the COVID-19 pandemic in all US states. For this objective, the number of confirmed cases of COVID-19 were obtained from the website maintained by the US Centers for Disease, and Prevention and Control (CDC) and R_t_ was calculated for each US state over time [6]. These values were used to train GTNN and NGNN models which were then asked to perform a simultaneous prediction of R_t_ for each state in the US. The efficiencies of the two models were compared with one another and with the results obtained using a baseline deterministic recurrent neural network (Long short-term memory [LSTM]) model.

This manuscript is structured as follows: Section 2 provides a brief explanation of previously published reports in which GNN was used to predict time series and pandemic-related data; Section 3 includes several basic models of graph theory, R_t_, and GNNs; Section 4 describes the different steps used to develop the predictive models; Section 5 documents the experimental methods, performance metrics, and outcomes and finally, Section 6 discusses the limitations of this study.

## 2. Literature Review

The first cases of COVID-19 in China were officially reported on 8 December 2019. This was followed shortly thereafter in January 2020 by the first confirmed case of COVID-19 in the USA [2]. By mid-February to March 2020, COVID-19 was recognized as a pandemic that affected all 50 states in the US, heralding unprecedented consequences [2]. On 8 December 2020, the CDC reported 285,351 COVID-19-related deaths and 15,208,638 confirmed cases in the US [6]. Given this dire situation, an accurate prediction of the dynamics of the pandemic may have a significant social and economic impact on the US [7]. GNN is one of the most accurate prediction methods in current use; several publications have already reported results using this method to evaluate time series data for pandemics.

Zheng et al. [8] proposed a hybrid spatio-temporal model that combined a susceptible-exposed-infectious-recovered (SEIR) within the framework of a recurrent neural network (RNN). This study featured a graph structure that included: (1) geographic neighbor effects (i.e., an edge feature) and (2) local temporal infection trends at the nodes. The study applied SEIR data at each node and RNN to each edge to achieve both efficiency and accuracy in model training and predictions [8]. The study used confirmed COVID-19 case data from all US states to predict anticipated case numbers one day and seven days in advance. The authors concluded that the hybrid spatio-temporal model outperformed standard RNN, SEIR, and autoregressive integrated moving average (ARIMA) models.

Nandini et al. [9] used graph theory and determined topological and thermodynamic entropy measures to compare the severity of the 1918 Spanish flu and the COVID-19 pandemic. The study considered several trees, including Cayley trees, Christmas trees, pandemic trees, and the corona products of Christmas trees and paths to obtain the topological indices. By calculating topological indices and thermodynamic entropy, Nandini et al. [9] clearly represented the severity of the COVID-19 pandemic compared to the 1918 Spanish flu. The networks, presented by Nandini et al. [9], can be used to develop accurate graph neural networks to predict the epidemiological dynamics of the COVID-19 pandemic.

Panagopoulos et al. [10] used a GNN to investigate the impact of human mobility on the geographical distribution of COVID-19 cases. The GNN model developed in this study used data from four European countries; the nodes represent the individual regions within each country, and the edges correspond to population movements between these regions. The model was used to predict the number of confirmed COVID-19 cases three days, seven days, and 14 days in advance. The authors concluded that the GNN model developed in this study outperformed traditional LSTM, ARIMA, and prophet models.

Cao et al. [11] focused on multivariate time series forecasting techniques to analyze a set of time series data points as a unified entity. The study proposed the use of a spectral temporal graph neural network (StemGNN) model to forecast the number of confirmed COVID-19 cases. StemGNN modeled both temporal dependencies (by applying a discrete Fourier transform) and inter-series correlations (by applying a graph Fourier transform) in the spectral domain [11]. Inputs included time series data from 25 countries from 22 January to 10 May 2020. The model was used to predict the number of confirmed cases of COVID-19 7, 14, and 28 days in advance.

La Gatta et al. [12] focused on mobility data and proposed a model that would identify traditional epidemiological parameters, including recovery and contact rates. To develop this model, LSTMs were combined with graph convolutional neural networks with both temporal and spatial features. The model was used to forecast the dynamics of COVID-19 in Italy from 24 February to 5 May 2020.

Kapoor et al. [13] developed a forecasting model that used GNN and mobility data to predict COVID-19 cases. In the proposed large-scale spatio-temporal graph, the spatial edges indicated the movement of a target population based on inter-region connectivity, the nodes indicated the region-level population movement, and the temporal edges represented node features over time [13]. The authors applied the model to a US county-level COVID-19 dataset and compared the results with those obtained using traditional LSTM and ARIMA models. Finally, Shah et al. [14] emphasized the importance of early contact-tracing to combat the COVID-19 pandemic and used GNN to locate patient zero (i.e., the source of an epidemic).

## 3. Basic Models

In this section, the basics of graph theory, functional connectivity, R_t_, and GNN are presented.

### 3.1. Graph Theory

Graph theory and network analysis have been used to address problems in various fields and have provided models that have been used to evaluate electrical power infrastructure, transportation systems, big data environments, social networks, biological neural networks, and complex brain networks [15]. In this theory, a graph consists of nodes that are linked by edges. Graph edges are defined as unweighted direct, weighted indirect, weighted direct, or unweighted indirect. These designations provide direct or indirect focus on the flow of information; the terms unweighted and weighted reflect the emphasis placed on the strength of their connections. The following eight steps explain the pipeline used to develop a functional network using graph theory:

Step 1. Defining the nodes of the network. Nodes can be changed based on the objective of the network.

Step 2. Preprocessing the time series data. The time series data needs to undergo preprocessing to remove noise and artifacts. Different preprocessing methods can be used, including Empirical Mode Decomposition (EMD). EMD is most suitable for processing non-stationary and non-linear data represented by a COVID-19 time series. EMD decomposes time series data into a finite number of multiple oscillatory modes called intrinsic mode functions (IMFs) [16]. For a given time series dataset, *x(t),* the process of EMD includes [17] the following:

- Identification of all extreme values of *x(t).*
- Interpolation between all local maxima and minima to create upper and lower envelopes, i.e., *e_max_(t)* and *e_min_(t)*.
- Computation of the average of these envelope values using the equation *m(t)* = [*e_max_(t)* + *e_min_(t)*]/2.
- Extraction of details using the equation *h(t)* = *x(t)* − *m(t)*,
- Iteration on the residual *r(t)* = *x(t)* − *c(t)*.

As summarized in Equation (1) *x*(*t*) can be decomposed into n IMFs and a residue using EMD:

(1) $$x(t)=\sum_{i=1}^{n}c_{i}(t)+r_{n}(t)$$

where *c* (*t*) represents IMF and *r (t)* residual.

Step 3. Defining the edges: In this model, the edges represent links and connections between nodes and display different patterns of functional or structural connectivity. When representing functional connectivity, the edges indicate the highly correlated time series of the nodes [18].

Step 4. Computing the connectivity matrix: The connectivity or adjacency matrix includes information concerning the connectivity patterns of the nodes. In this matrix, the connectivity is explained by an N × N symmetric matrix, in which the columns (j) and rows (i) are represented by nodes, and all matrix entries (a_ij_) represent edges [18].

Step 5. Converting the connectivity matrix into a binary matrix: Matrix binarization is used to develop an unweighted unidirectional matrix from the data in the adjacency matrix [15]. For this purpose, a threshold value is identified as a first step. If the correlation between two nodes in the connectivity matrix exceeds the threshold, the value of the edge corresponding to those nodes is one, otherwise zero.

Step 6. Selecting the threshold value: This value is used to simplify the complexity of the network by removing insignificant or weak edges.

Step 7. Selecting and applying a functional connectivity measurement: Various measurements can be used to calculate functional connectivity, including correlation, magnitude squared coherence, phase-locking value, mutual information, and transfer entropy [18].

Step 8. Constructing the network: The network can be constructed by following the aforementioned steps.

### 3.2. Effective Reproduction Number (R_t_)

The effective reproduction number, or R_t_, is defined as “*the expected number of new infections caused by an infectious individual in a population where some individuals may no longer be susceptible*” [3]. Calculation of R_t_ can provide information on dynamics of COVID-19 transmission during specific time-steps [19]. To eliminate virus transmission, R_t_ will need to be reduced to 0. However, to bring the pandemic under control, the R_t_ must be less than 1 [4]. Therefore, estimates of situation- and time-specific values of R_t_ can help us to understand the extent of pathogen transmissibility at a given point in time. Among the different methods that have been developed to estimate R_t_, we selected the instantaneous reproductive number method developed by Cori et al. [4] which is as follows:

(2) $$R_{t}=\frac{I_{t}}{\sum_{s=1}^{t}I_{t-s}w_{s}}$$

In Equation (2), *w_s_* is the generation interval, which has been defined as “the time between the infection time of an infected person and the infection time of her or his infector”, and *I_t_* is defined as the number of incidents of infections on day *t* [20].

The generation interval (*w_s_*) used here was taken from Nishiura et al. [21] who reported mean serial intervals of 2.9 days (95% credible interval [CrI], 1.9–4.9) and 4.7 days (95% CrI, 3.7–6.0). The Excel file of EpiEstim package was borrowed from Cori et al. [4] to determine R_t_ (Please refer to https://github.com/RezaDavahli/Graph_neural_networks for models and input data; accessed on 10 March 2021).

### 3.3. Graph Neural Networks (GNNs)

GNNs have been used for various applications, including the development of computer vision, natural language processing, and chemistry [12]. However, one of the main applications of GNN is time series forecasting. In GNNs, information from node connections is paired with input from the node signal to inform the hidden state of the input layer [13]. GNNs utilize a combination of graphs and structures associated with convolutional neural networks. Specifically, graph convolutional networks are modifications of standard convolutional neural networks that can be used to detect low-level features from data based on node characteristics together with their neighboring topology and aspects [12]. These low-level features can be used for various tasks, including node prediction, node labeling, and edge prediction. In this study, models were developed based on graph convolutional networks. In the graphic representation, G = (V, E) where E and V are the edges and nodes sets, respectively, and A is its associated adjacency matrix; the *H^(l)^* layer is developed recursively as per the following equation [22]:

(3) $$H^{\left(l+1\right)}=f\left(H^{\left(l\right)},A\right)=\sigma \left({Ď}^{-\frac{1}{2}}.Ȃ.{Ď}^{-\frac{1}{2}}.\;H^{\left(l\right)}.W^{\left(l\right)}\right)$$

where, Ȃ = *A* + I (I is the identity matrix), *σ* is activation function, *W^(l)^* is the weight matrix for the *l*-th layer, Ď represents the diagonal node degree matrix of Ȃ.

## 4. The Pandemic Prediction Model

We considered two types of graphs while developing these models. To generate an NGNN, we linked the neighboring states together and constructed the graph. To generate a GTNN, we constructed a graph based on principles of graph theory and functional connectivity. In this latter graph, nodes represent states in the US. By considering the spread of SARS-CoV-2 across the US as a very complex network, graph theory will help us to analyze the dissemination of the virus by generating mathematical relationships that provide links between various states.

**Figure 1:**
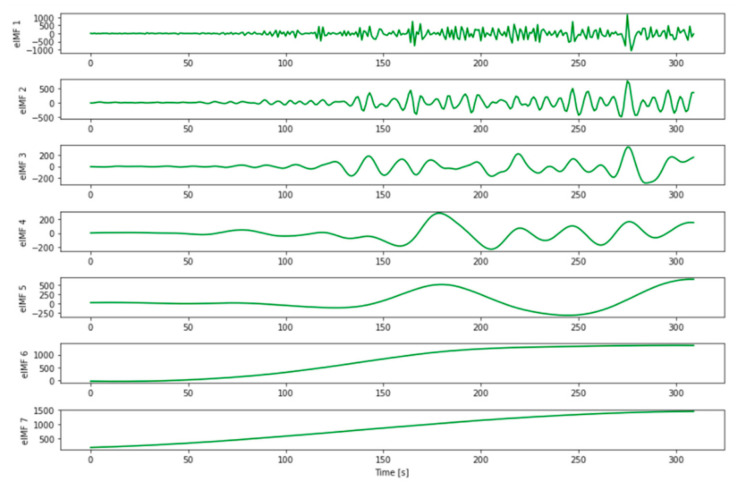
IMFs generated for Alabama.

For this purpose, time series data were collected for all states in the US from the website maintained by the CDC [6]. EMD was used for preprocessing the collected time series data on confirmed cases of COVID-19. We used the PyEMD library for the Python-driven implementation of EMD [23]. As a result of EMD preprocessing, the dataset from each state was divided into seven or eight IMFs. For example, IMFs associated with the state of Alabama are shown in Figure 1.

**Figure 2:**
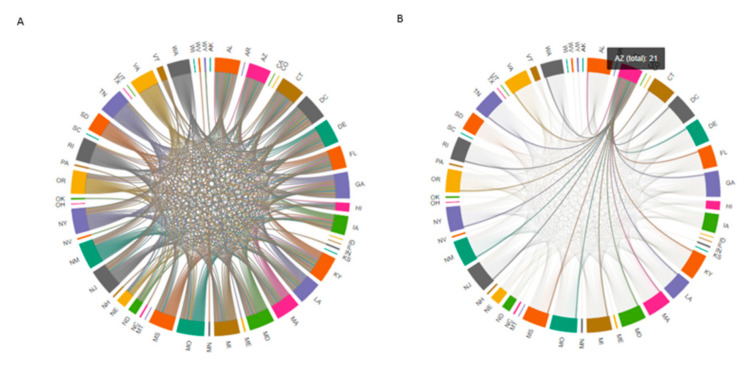

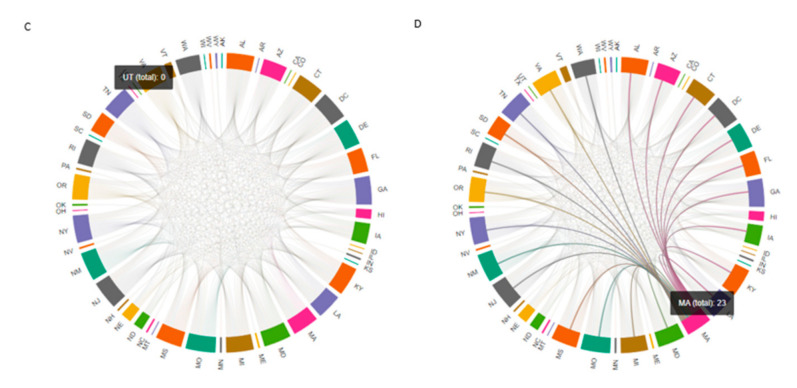
The COVID-19 correlation network for all US states (**A**), Arizona (**B**), Utah (**C**), and Massachusetts (**D**) (Please refer to https://github.com/RezaDavahli/Graph_neural_networks for live figure; accessed on 10 March 2021).

We first removed the highly-oscillating IMFs, including IMF 1 (which was oscillated daily) and IMF 2 (which was oscillated on a near weekly basis). We then combined the remaining IMFs and used them to construct smoothed time series data for all states. Pearson’s correlation coefficients (r) were calculated between time series from all states. Because values for “r” were high when calculated in this manner, we computed Pearson’s correlation coefficients between percent changes in time series to make certain that the correlation coefficients represented actual connections between time series in all US states. The results yielded a symmetric correlation matrix C_ij_ (size 51 × 51), in which an element in position i, j indicated a correlation between percent changes in the time series of states i and j. We introduced 0.3 as a threshold value and developed a binary matrix that maintained the strongest links between the time series of different states and removed the weakest connections. The final results were used to construct the COVID-19 correlation network as shown in Figure 2. As an example of the interpretation of these findings, the COVID-19 time series for the state of Arizona correlated with those of 21 other states; by contrast, the time series for the state of Utah did not correlate with those from any other state evaluated.

The results from Figure 2 indicates that the spreading pattern of SARS-CoV-2 in some states is highly correlated with each other. Therefore, correlated states have considerable impacts on each other in terms of the spreading pattern of COVID-19. For example, the behavior of COVID-19 in Arizona has considerable impacts on the virus’s spreading pattern of the virus in Florida, Georgia and 19 other states (please refer to https://github.com/RezaDavahli/Graph_neural_networks for live figure; accessed on 10 March 2021).

**Figure 3:**
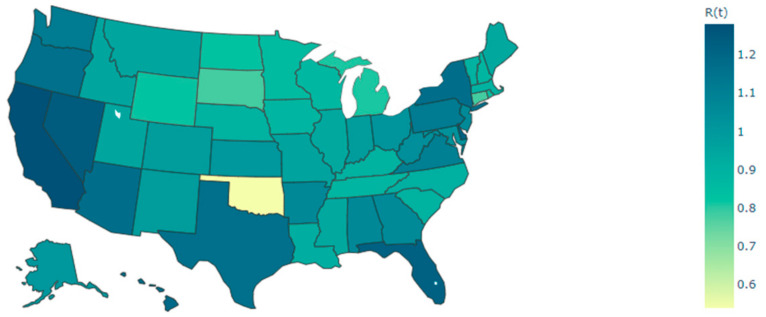
R_t_ determined for all states in the US on 26 November 2020.

GTNN and NGNN models were used to predict values for R_t_ in all states of the US. The number of confirmed COVID-19 cases from 22 January to 26 November 2020, was extracted from the CDC website. The Excel file of the EpiEstim package was borrowed from Cori et al. [4] to calculate R_t_. For example, R_t_ for all states of the US on 26 November 2020, is shown in Figure 3.

**Figure 4:**
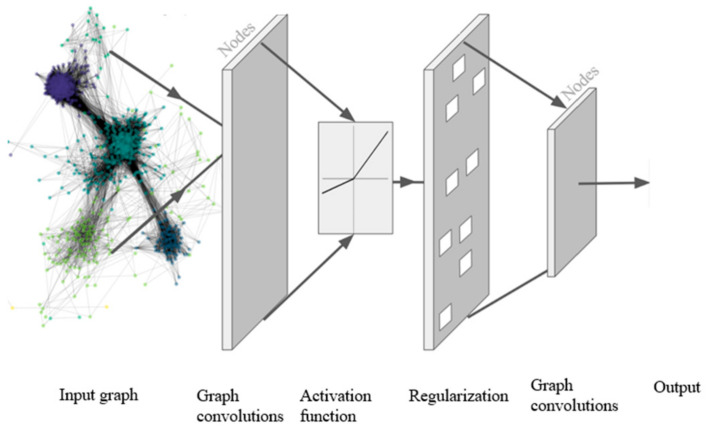
Graph Convolutional Networks [5].

After calculating R_t_, the GTNN and NGNN models were trained with this information. Information flow in these models proceeds as follows:

- Passing and receiving information between connected nodes (message passing);
- Aggregating and embedding of connected nodes;
- Passing information to the activation function; and
- Applying regularizations such as dropout, as shown in Figure 4 [5].

## 5. Experimental Study

In this section, we describe the evaluation of the GTNN and NGNN models using the R_t_ dataset. The results from each model were then compared with one another and with those obtained using a baseline LSTM model to understand their predictive accuracy.

### 5.1. Experimental Design

**Figure 5:**
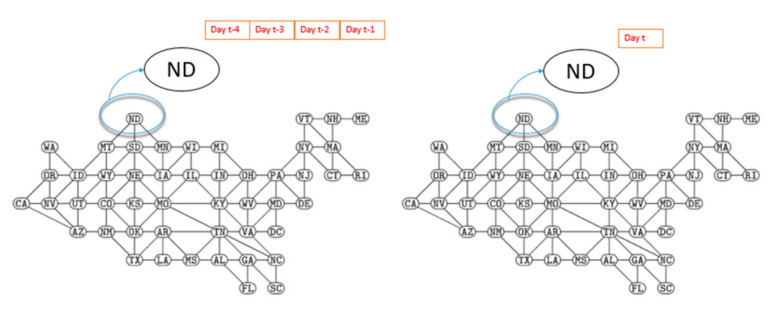
Training dataset. The node features of the state of North Dakota (ND) are shown.

The performance of these models was evaluated with the datasets that included R_t_ values reflecting conditions from 22 January through 26 November 2020. In both of these models, the individual states were identified by nodes. For the NGNN model, the edges were connections between neighboring states; for the GTNN model, the edges represented functional connectivity. Node features include R_t_ of time steps; no specific edge features were identified. For the training and testing datasets, we used R_t_ values for each state calculated for the previous four days (i.e., node features) to train and predict the value R_t_ for each state on the day to follow (one day sliding window). This is shown for the NGNN model in Figure 5. It should be mentioned that we tried different time window and we reached a conclusion that training based on the previous four days provided the the best results. The datasets were divided into training and testing datasets. We used 98% of data for training (including a randomly-selected 75% for training and 23% for validation) and 2% for testing (data for seven days for testing from 20 November 2020 to 26 November 2020 was applied). The number of days selected for the testing period was adopted from Zheng et al. [8] in order to provide improved comparability of our results.

Therefore, the main task of these models was to predict future node features in all states based on those identified previously. The Pytorch [24] and PyTorch Geometric [25] libraries in Python were used to develop NGNN and GTNN models. We also utilized an ADAM optimizer [26]. Our models included three hidden layers labeled conv1, 2, and 3.

### 5.2. Performance Metrics

The percentile error of the models, calculated as the Symmetric Mean Absolute Percentage Error (sMAPE), was used to evaluate the performance of the model as per Equation (4) [27,28]:

(4) $$sMAPE_{i}=\;\frac{1}{T}\overset{T}{\underset{t=1}{\sum }}\frac{\left|y_{i,t}-\;{ŷ}_{i,t}\right|}{y_{i,t}+\;{ŷ}_{i,t}}$$

where *y_i,t_* is the real value in state *i* at time-step *t*, and *ŷ_i,t_* is the predicted value.

### 5.3. Performance Results

**Figure 6:**
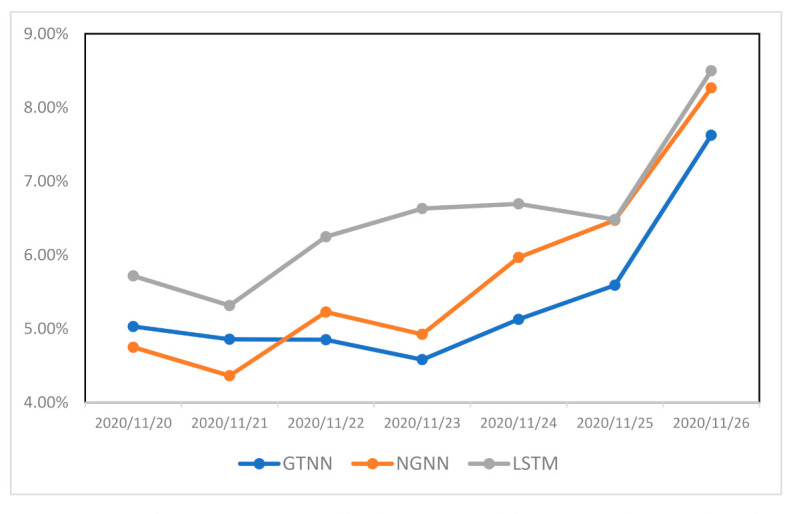
sMAPE for GTNN, NGNN, and baseline LSTM models over seven days as indicated.

To compare the performance of these models, we computed sMAPE for seven testing days as shown in Figure 6. For each time step, R_t_ values for four previous days were used to compute prediction data. Then, sMAPE was calculated by using equation #4 based on actual and predicted data. For example, R_t_ values from 16 November 2020 to 19 November 2020were used to predict R_t_ values on 20 November 2020. The average sMAPE of the GTNN model was 5.38%; the sMAPE values calculated for the NGNN and LSTM models were 5.71% and 6.51%, respectively. Comparing the efficiency of GTNN and NGNN indicates that the selection of graph (network) has a considerable impact on the accuracy of the predictive GNN model. Therefore, by selecting efficient networks, we can have accurate predictive models.

**Figure 7:**
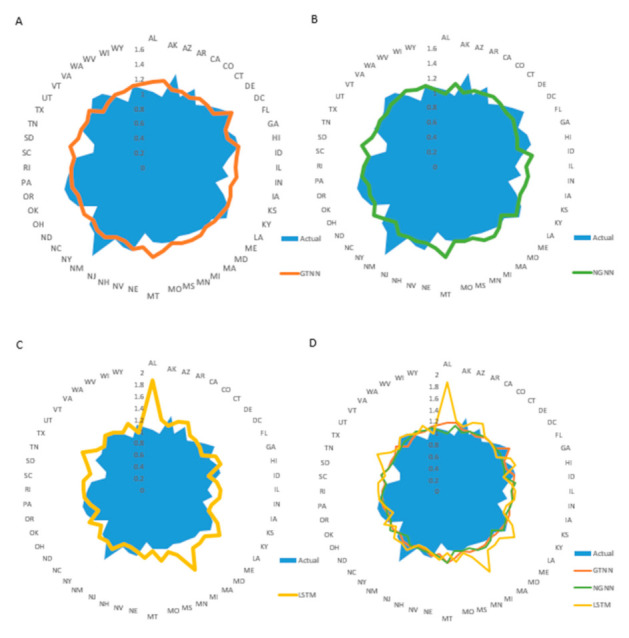
Actual and predicted values for R_t_ for all states on 23 November 2020. Shown are values predicted using the GTNN (**A**), NGNN (**B**), and LSTM (**C**) models. Superimposed results from all models are shown in (**D**).

We also represented the forecasting performance of the GTNN, NGNN, and LSTM models for all US states on 23 November 2020 (Figure 7). As shown, both GTNN and NGNN outperformed the baseline LSTM model.

**Figure 8:**
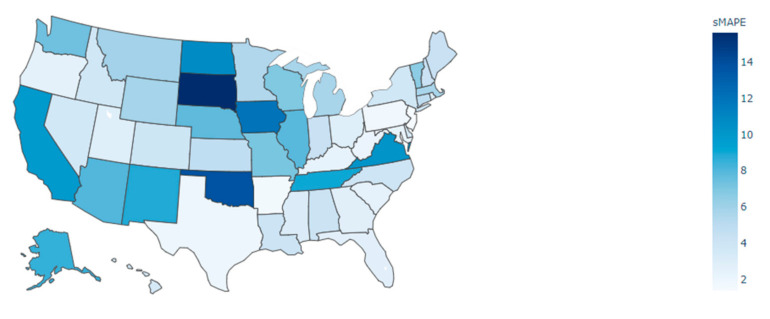
Average sMAPE for all states in the US using predictions from the GTNN model.

We also calculated the average sMAPE predicted by the GTNN model for all US states as shown in Figure 8. As shown, New Jersey, Arkansas, Pennsylvania, and Texas displayed minimum values of sMAPE. By contrast, South Dakota, Oklahoma, Iowa, and North Dakota displayed the maximum values of sMAPE over the seven-day period. (Please refer to https://github.com/RezaDavahli/Graph_neural_networks for models and input data; accessed on 10 March 2021).

## 6. Limitations

One of the limitations of this study was that we did not consider the impact of individual states on one another in the baseline model. Some of the literature that we reviewed did consider the potential for interactions between states, notably in the LSTM baseline model. Another limitation of the current study is that we did not consider R_t_ values once the vaccines were widely distributed or in the case of spreading of variants of the virus. Because we started working on this study before the availability of COVID-19 vaccination and new variants of the virus, we did not consider these factors’ impact on the predicted R_t_.

A third limitation is related to using effective reproductive numbers. To better capture the dynamics of the pandemic, we decided to predict R_t_ rather than total number of COVID-19 confirmed cases or total number of deaths. However, this decision had some restrictions. First, R_t_ can be computed from different methodologies, which each one of them can give a different estimate. Second, computing R_t_ requires assuming the generation interval. To avoid biases in R_t_ calculation, it is essential to estimate and specify the generation interval distribution accurately.

This study’s final limitation is related to the efficiency of developed models for different US states. As shown in Figure 8, the displayed average sMAPE values represent the differences between our model’s forecast and actual values for the seven days of the testing period. Because developed models trained based on previous days, it is possible that for a different testing period, the outcome measure would be different for states of the US.

## 7. Conclusions

Our objective was to develop a methodology that can be used to predict the dynamics of the pandemic in the US. The two main elements of this methodology included a predictive model and an indicator that could be used to assess the state of the pandemic at any given time. We considered the effective reproduction number (R_t_) as a strong indicator of the state of the pandemic. To bring the pandemic under control, R_t_ must be less than 1; the pandemic will be effectively eliminated once R_t_ is at or near zero. Therefore, this number can be used as an indicator of the state of the pandemic at any given time. We selected GNN models as predictive methods and as a means to consider the impact of US states on one another. Also, this method is very effective at forecasting using time series data. We trained the GNN models with historical patterns of R_t_ and compared the results obtained with those from a baseline LSTM model. The results indicated that the GNN models outperformed the LSTM models.

## Data Availability

The data presented in this study are openly available in https://github.com/RezaDavahli/Graph_neural_networks, accessed on 10 March 2021.
